# Benchmarking beta-diversity measures and transfer functions for sedimentary ancient DNA

**DOI:** 10.1093/ismeco/ycaf230

**Published:** 2025-12-06

**Authors:** Tristan Cordier, François Keck, Anders Lanzén

**Affiliations:** NORCE Climate & Environment division, Norwegian Research Centre & Bjerknes Centre for Climate Research, Jahnebakken 5, Bergen 5007, Norway; Department of Evolutionary Biology and Environmental Studies, University of Zurich, Winterthurerstrasse 190, CH-8057 Zürich, Switzerland. Eawag, Swiss Federal Institute of Aquatic Science and Technology, Department of Aquatic Ecology, Überlandstrasse 133, CH-8600 Dübendorf, Switzerland; AZTI, Marine Research, Basque Research and Technology Alliance (BRTA), Herrera Kaia, Portualdea z/g, 20110 Pasaia, Gipuzkoa, Spain; IKERBASQUE - Basque Foundation for Science, Plaza Euskadi 5, 48009 Bilbao, Bizkaia, Spain

**Keywords:** sedimentary ancient DNA, beta-diversity, transfer functions, machine learning, paleoecology, paleoceanography, paleolimnology

## Abstract

Analyzing past ecosystems can improve our understanding of the mechanisms linking biodiversity with environmental changes. Sedimentary ancient DNA (*sed*aDNA) opens a window to past biodiversity, beyond the fossil record, that can be used to reconstruct ancient environments and ecosystems functions. To this end, modern biodiversity and environmental conditions are used to calibrate transfer functions, that are then applied to past biodiversity data to reconstruct environmental parameters. Doing this with *sed*aDNA can be challenging, because ancient DNA is often obtained in limited quantities and fragmented into smaller molecules. This leads to noisy datasets, with a low alpha diversity relative to modern DNA, patchy taxa detection patterns and/or skewed relative abundance profiles. How this affects beta-diversity measures, and the performance of transfer functions remain untested. Here we simulated ancient DNA reads counts matrices from synthetic and empirical datasets, and tested 464 combinations of counts transformations (n = 13), beta-diversity indices (n = 16), and ordinations methods (n = 4), and assessed their performance in (i) separating the ecological signal from the noise introduced by DNA degradation and in (ii) predicting ground-truth environmental conditions. Our results show that commonly used workflows in DNA-based community ecology studies are sensitive to the noise associated to ancient DNA signal. Instead, combinations of methods that include more recent ordination methods proved robust to ancient DNA noise and produced better transfer functions. Our study provides a framework for designing postprocessing workflows that are better suited for *sed*aDNA studies.

## Introduction

Documenting past environments and associated biodiversity is essential to improve our understanding of the dynamics of natural ecosystems over geological timescales. Current environmental reconstructions in paleoceanographic or paleolimnological studies commonly rely on transfer functions calibrated on microfossil assemblages [1]. For instance, assemblages of planktic foraminifera have been used to reconstruct Atlantic sea-surface temperatures [2, 3], dinoflagellate cysts for Arctic sea ice [4], and diatoms for lake salinity [5, 6], trophic status [7] water quality [8] and climatic variables [9]. All of these methodologies rely on the ecological niche concept [10], which defines a theoretical multi-dimensional space where the dimensions represent environmental conditions under which a species can survive and reproduce. In practice, transfer functions exploit this principle by assuming a consistent and quantifiable relationship between species assemblages and environmental variables. Thus, species composition becomes a proxy for reconstructing past environmental conditions, based on the idea that taxa respond predictably to environmental gradients through their realized niches. Current transfer functions methodologies are limited to taxa that fossilize and can be identified in the sediment record. This means that only a fraction of an ecosystem’s biodiversity can be considered, because soft-bodied taxa are not preserved in the sediment.

High-throughput sequencing of sedimentary ancient DNA (*sed*aDNA) is a powerful tool to study long-term biodiversity changes beyond the fossil record, as it provides direct molecular evidence of past communities preserved in environmental archives such as lake or marine sediments [11–14]. Over the last decade, *sed*aDNA datasets have been obtained at centennial timescales in lakes [15–17] and at glacial/interglacial (i.e. 10–200 000 years ago, [18, 19]) and even million-year [20, 21] timescales in marine ecosystems. These *sed*aDNA datasets thus open the path to leverage entire biodiversity datasets to reconstruct ancient environmental conditions. However, interpreting *sed*aDNA data remains challenging due to several biological and technical limitations. Among these, whether ancient DNA molecules from different taxa are evenly preserved in the sediment over time is not yet understood or documented, although sediments seem to present favorable physicochemical features for long-term DNA preservation [22, 23]. Such an uneven DNA preservation would result in missing taxa or patchy detections in *sed*aDNA datasets (i.e lower alpha diversity due to false negatives), and/or in skewed relative abundances profiles across samples (i.e. noisy beta-diversity). How this affects ecological inferences from beta-diversity patterns analysis, or environmental reconstructions via transfer functions, remain untested.

When analyzing biodiversity data obtained from DNA-based methods (i.e. metabarcoding or shotgun metagenomics), one needs to process the DNA read counts matrices, i.e. the community composition, via successive steps prior beta-diversity analysis or transfer function calibration. First, DNA read counts need to be transformed (or normalized, usually using relative abundances or center log ratio), to compensate for the compositional nature of the data [24–26]. Then, these transformed counts are used to calculate pairwise community dissimilarities using a beta-diversity index [27, 28] (usually using Bray–Curtis or Euclidean distances). Finally, this dissimilarity matrix is used as input for an ordination method (usually a Non-metric Multidimensional Scaling, NMDS or Principal Coordinate Analysis, PCoA) to visualize beta-diversity patterns in a two-dimensional space [28]. While some counts transformation, beta-diversity indices and ordination methods have been developed decades ago, some more recent methods have been proposed to improve beta-diversity analyses or extract new ecological signal from high-dimensional datasets (Tables S1–S3), such as the ones produced with high-throughput sequencing technologies. For instance, the robust centered log ratio count transformation overcomes the need for adding a pseudocount to the matrix as it is the case with standard center log ratio transformation [29], and the wrench count transformation implements an empirical Bayes normalization approach to adjust for nonconstant variance across samples [30]. Some beta-diversity indices now account for the phylogenetic signal extracted from DNA sequences to quantify community dissimilarities (i.e. UniFrac, [31] and PINA, [32]) or account for the taxa putative interaction patterns rather than only presence/absence (i.e. TINA, [32]), capturing new ecological dimensions from DNA-based datasets (Table S2). Newer ordination methods for high-dimensional data embedding (Table S3), such as t-Distributed Stochastic Neighbor Embedding (tSNE, [33]) and Uniform Manifold Approximation and Projection (UMAP, [34]) have recently proved useful for the analysis of beta-diversity patterns from DNA-based datasets [35, 36]. How the choice of the method for each of these of processing steps affect the results of a beta-diversity analysis has received substantial attention, but have mostly focused on either the effect of counts transformation [37–40], the effect of beta-diversity index [27] and the effect of the ordination method [35, 36]. No systematic benchmark of the effect of the multitude of possible combinations of these methods on the beta-diversity analysis has been carried out. Similarly, no benchmark currently exists on the effect of these processing steps on the performance of sample classification using supervised machine learning methods, or on the performance of transfer functions for environmental reconstructions with *sed*aDNA.

**Figure 1:**
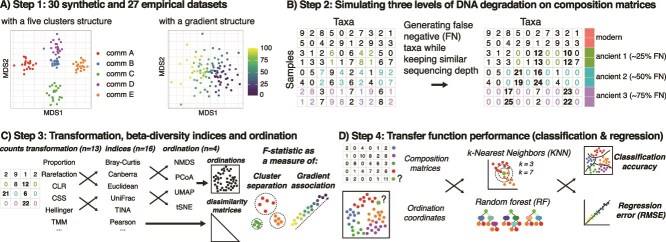
Overview of the approaches for data simulations and analysis. (A) We produced 30 composition matrices using the splatter R package (synthetic datasets) and collected 27 environmental genomics datasets (empirical datasets, Table S1). (B) Assuming that DNA degradation would generate false negatives taxa detections, we simulated three levels of false negative taxa, while keeping a similar sequencing depth (see methods for details). (C) We produced 464 unique combinations of count transformation, beta-diversity indices and ordinations, and used the F-statistic of PERMANOVA models to measure their performance in separating clusters and find association with ecological gradients. (D) We assessed the performance of these combinations of methods in producing accurate transfer functions, i.e. predicting correctly the cluster of origin and value on an ecological gradient for simulated ancient DNA samples.

Here we simulated ancient DNA matrices from both synthetic and empirical community data to systematically benchmark 464 combinations of count transformation techniques, beta-diversity indices, and ordinations methods (Fig. 1). We evaluated their performance in (i) capturing the true ecological structure while compensating for technical noise introduced by the ancient DNA signal through beta-diversity patterns and (ii) in producing accurate supervised machine learning models and transfer functions. Our goal was to identify the combinations of methods that maximize ecological signal and transfer function performance, even under the challenging constraints of *sed*aDNA data, such as possibly large rates of taxa dropout (or false negatives) due to DNA degradation over time.

## Materials and methods

### Synthetic community datasets

We used the *splatter* R package v1.28.0 [41] to simulate 30 composition matrices comprising 100 samples and 400 taxa, to keep the computational needs reasonable. Simulations were run both in a cluster (for community typing, n = 5 clusters) and in a path (for ecological gradient) setting, resulting in a total of 60 composition matrices (Fig. 1A). For each simulation run, we used uniform distributions to generate a unique set of parameters that control the hierarchical probabilistic models from which the DNA read counts are drawn for populating composition matrices. Details of these simulations are available in the scripts deposited on the GitHub repository (https://github.com/trtcrd/Bench_sedaDNA), that allows reproducing the simulations as well as the downstream analysis.

### Empirical community datasets

We compiled 27 environmental genomics datasets (Table S1) of contrasted taxa (from bacteria to protists), in contrasting habitats (from pelagic to benthic and soil) and climates (from tropical to polar). We retrieved the composition matrices and associated metadata as they were produced in the respective studies. For datasets with a discrete variable (cluster analysis, n = 21), we balanced the number of samples per variable class to keep a fair comparison of classification performance. For datasets with a continuous variable (gradient analysis, n = 10), we kept all the original samples. To keep the computational needs of our benchmark reasonable, we reduced the number of samples to a maximum of 125 (Table S1). We also reduced the number of taxa to 1000–4000 taxa, by subtracting the same number of DNA reads across the entire composition matrices in order to keep the community structure. Finally, we kept only the samples with at least 20 taxa.

### Simulating ancient DNA signal

The expected signal of ancient DNA on community profiles is to have various levels of false negative taxa, i.e. taxa from which the DNA is not sequenced because it is not well preserved in the sediment record. Because the sequencing effort is a technical parameter, the DNA reads that are not dedicated to these false negative taxa would randomly cover the taxa from which DNA molecules are effectively present in the sample, and preferentially the most abundant ones. Under this rationale, we simulated three effect sizes of ancient DNA signal, by generating ~25, 50, and 75% false negatives taxa (allowing a small random noise around these values), randomly selected across the composition matrices (Fig. 1B). This was done evenly across each variable class for community clusters or randomly along the ecological gradient, in order to have an even number of samples for each ancient DNA effect size (i.e. 25% of samples at 0, 25, 50, 75% of false negatives). The reads subtracted from the false negatives were then allocated to a random selection of the 10% most abundant taxa of a given sample, to preserve the sequencing depth across the composition matrices.

### Statistics

We produced a total of 464 (synthetic datasets) and 396 (empirical datasets) combinations of count transformation techniques, beta-diversity indices and ordinations methods (Fig. 1C, Tables 1–3). For synthetic datasets, we mapped real plankton sequences obtained from a previous study [42] to the simulated taxa, using the same ranked relative abundances (i.e. most abundant plankton sequence from the study was mapped to the most abundant taxa in each synthetic dataset), allowing us to test combinations that include sequence-centered transformation techniques and beta-diversity indices (Table 2). We used the F-statistic of permutational analysis of variance (PERMANOVA) models as a measure of performance in cluster separation or gradient association, using the *adonis2* function of the *vegan* R package v2.6–6.1 [43]. This was measured from both samples pairwise dissimilarity matrices and from an Euclidean distance matrix extracted from the samples coordinates on the ordination’s embeddings. We also measured the changes in betadispersion, i.e. the multivariate variance, as a function of increasing ancient DNA effect size (from modern DNA to ancient DNA with 75% of false negative taxa) using the F-statistic output of the *betadisper* function of the vegan R package.

**Table 1:** Count transformation methods investigated in this study.

Abbreviation	Description	Reference
prop	Proportion: Counts in each sample are divided by the sample’s total count to calculate relative abundances.	
rarefy	Rarefaction: Subsamples each sample without replacement to an equal, fixed count to standardize sequencing depth.	[44]
hellinger	Hellinger transformation: Takes the square root of relative abundances to reduce the influence of highly abundant taxa.	[45]
chi.square	Chi-square transformation: Scales data by dividing by sample totals and taxa sums, then adjusting by the matrix total’s square root. When paired with Euclidean distance, approximates Chi-square distance used in correspondence analysis.	[46]
CSS	Cumulative Sum Scaling: Normalizes by computing a scaling factor per sample based on counts up to a data-driven threshold, reducing bias from highly variable abundant taxa to improve cross-sample comparability.	[47]
clr	Centered Log-Ratio: Transforms positive data by taking log ratios centered on the geometric mean, reducing compositional bias. Zeros are typically handled by adding a small pseudocount before transformation.	[48]
rclr	Robust Centered Log-Ratio: Similar to clr but accommodates zeros without pseudocounts by centering on the geometric mean of observed (nonzero) features, keeping zeros as zero.	[29]
VST	Variance Stabilizing Transformation: Uses modeled mean-dispersion relationships to transform normalized counts, producing data with approximately constant variance across means.	[49]
TMM	Trimmed Mean of M-values: Normalizes data by weighting log-fold changes (M-values) with inverse variance estimates to adjust for compositional differences between samples.	[50]
TMMwsp	Trimmed Mean of M-values with Singleton Pairing: Extension of TMM designed for sparse data with many zeros, pairing singleton positive counts across samples to stabilize normalization.	[51]
wrench	Wrench Transformation: An empirical Bayes method estimating sample-specific scaling factors using group information and a hurdle log-normal model to normalize sparse, zero-inflated data and correct compositional bias.	[30]
GMPR	Geometric Mean of Pairwise Ratios: Normalizes zero-inflated data by calculating size factors based on medians of pairwise abundance ratios, robust to zeros and outliers, ideal for sparse microbiome counts.	[52]
kmer	k-mer Decomposition: Breaks sequences into overlapping k-length substrings (k-mers), then counts and normalizes based on k-mer frequency	[53]

**Table 2:** Beta diversity metrics investigated in this study.

Abbreviation	Metric	Description	Reference	R package
Euc	Euclidean	Calculates the straight-line distance between two points in multi-dimensional space.		vegan
Bray	Bray–Curtis	Measures dissimilarity based on the sum of minimum shared abundances relative to total abundances.	[54]	vegan
Canberra	Canberra	Emphasizes relative differences by weighting smaller values more heavily in the distance calculation.	[55]	vegan
Kulczynski	Kulczynski	Computes the average ratio of minimum to total abundances, highlighting shared low-abundance taxa.	[56]	vegan
Horn	Horn	Quantifies pairwise overlap in taxa abundances, adjusted by sample-specific concentration indices.	[57]	vegan
Gower	Gower	Handles mixed data types by averaging scaled differences across all variables, both numeric and categorical.	[58]	vegan
altGower	Alternate Gower	Variant of Gower distance with adjusted weighting between categorical and numeric variables.	[59]	vegan
Chao	Chao	Estimates similarity while accounting for unseen species pairs to correct undersampling bias.	[60]	vegan
Pearson	Pearson	Distance derived from Pearson correlation coefficient, capturing linear association between samples.	[61]	factoextra
Spearman	Spearman	Distance derived from Spearman rank correlation, capturing monotonic relationships between samples.	[62]	factoextra
RF	Random Forest Proximity	Measures similarity based on the frequency two samples fall into the same terminal nodes in Random Forest classification.	[63]	randomForest
TINA	Taxa Interaction-Adjusted, weighted	Calculates weighted average similarity of pairwise taxa interactions based on abundances.	[32]	See paper github
PINA	Phylogenetic Interaction-Adjusted, weighted	Calculates weighted average similarity of pairwise taxa phylogenetic relationships.	[32]	See paper github
UniFrac	Fast UniFrac, weighted	Measures phylogenetic distance as the fraction of branch length unique to each community, weighted by abundance.	[64]	phyloseq
gUniFrac05	Generalized UniFrac (α = 0.5)	Generalized UniFrac balancing presence/absence and abundance differences with α = 0.5.	[65]	gUniFrac
gUniFracVAW	Variance Adjusted Weighted UniFrac	Generalized UniFrac variant that adjusts weights to reduce variance effects and improve sensitivity.	[66]	gUniFrac

**Table 3:** Ordinations methods investigated in this study.

Abbreviation	Description	Use case	Reference	R package
PCoA	Principal Coordinate Analysis: Projects dissimilarity matrix (e.g. Bray–Curtis) into Euclidean space	Beta-diversity plots	[67]	ape
NMDS	Non-metric Multidimensional Scaling: Preserves rank order of dissimilarities into the ordination space	Non-linear ecological gradients	[68]	vegan
t-SNE	t-Distributed Stochastic Neighbor Embedding: Preserves local neighborhoods / manifold structure	Clustering or visualizing sample types, often in ML workflows	[33]	Rtsne
UMAP	Uniform Manifold Approximation and Projection: Preserves local neighborhoods / manifold structure	Clustering or visualizing sample types, often in ML workflows	[34]	umap

Then, we measured the performance of two supervised machine learning algorithms commonly used by microbial ecologists, namely k-nearest neighbors (KNN, [69]) and random forest (RF, [63]), in correctly classifying the samples (classification accuracy) or regressing the ecological gradients (root mean squared error, RMSE) using cross validation on each dataset. Gradients were normalized from 0 to 1 to keep the RMSE comparable across studies. Models were trained either on composition matrices (using the transformed DNA read counts of all taxa as features) or on ordination embeddings (using the samples coordinates in the two-dimensional space as features). We trained and tested the models using a 60–40 train-test split combined with repeated 10-fold cross-validation (10 repetitions) using the *caret* R package v6.0–94 [70], applied separately to each “modern DNA” and “ancient DNA” dataset.

We then measured the performance of KNN and RF-based transfer functions approaches (calibrated on composition matrices or ordination embeddings) in predicting the cluster of origin (transfer accuracy) or ecological gradient values (transfer RMSE) of the ancient DNA samples of each dataset, based on a model trained only on the modern DNA samples of that dataset (Fig. 1D). Finally, we aggregated the performance metrics of the top five transfer function approaches and used Tukey's Honest Significant Difference to test for differences in performance between KNN and RF-based transfer function approaches calibrated in either composition matrices or ordinations embeddings. Plots were created using the *ggplot2* R package v3.5.1 [71], and arranged with the *patchwork* R package v1.2.0 [72].

## Results

### Detecting clusters and gradients in distance matrices

**Figure 2:**
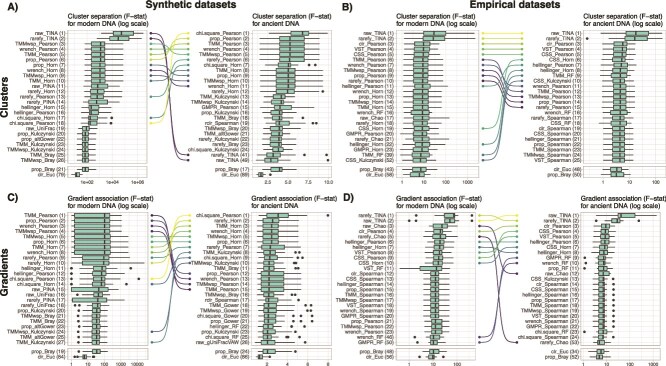
Performance of combinations of DNA read counts transformation and beta-diversity indices for cluster separation in (A) synthetic datasets and (B) empirical datasets, as well as of gradient association in (C) synthetic datasets and (D) empirical datasets. Performance was measured with the F-statistic of PERMANOVA models (higher is better). The left panels show the F-statistic distributions for modern DNA datasets, whereas the right panels show the F-statistic distributions for simulated ancient DNA datasets. Bumps charts connect identical combinations of methods between modern and ancient DNA datasets that are among the 10 best approaches. Numbers between brackets indicate the ranked position of the combinations of count transformation and beta-diversity indices based on the median F-statistic value.

Our results based on synthetic datasets show that the combinations of count transformation and beta-diversity indices that worked well for modern DNA corresponded, in general, to the ones that worked well for ancient DNA datasets (Fig. 2A, C, Tables S2–S3). However, the combinations that included the TINA index worked particularly well for detecting clusters in modern DNA, while they performed poorly for ancient DNA for synthetic datasets (Fig. 2B), but well for empirical datasets (Fig. 2D). For both clusters and gradients, the Chi-Square transformation combined with the Pearson dissimilarity index (i.e. 1—Pearson correlation between pairs of samples) resulted in the highest F-statistic across the 30 ancient DNA datasets, but ranked only 18th and 13th for cluster and gradient from modern DNA, respectively. This was followed by several combinations of methods that included the Horn (i.e. 1 minus the Morisita-Horn index) or the Pearson index, that also worked well for modern DNA datasets. Interestingly, for empirical datasets, the two combinations of methods that included the TINA index resulted in the highest F-statistic for both modern and ancient DNA and for both cluster separation and gradient association, followed by multiple combinations of methods that included either the Pearson or Horn indices (Fig. 2B, D, Tables S4–S5). For both synthetic and empirical datasets, the standard combinations of methods that are commonly used by environmental microbial ecologists (i.e. proportion and Bray–Curtis) or by human gut microbial ecologists (i.e. centered log ratio and Euclidean distance) were never among the best performing approaches for detecting clusters and gradients.

### Detecting clusters and gradients in ordinations

**Figure 3:**
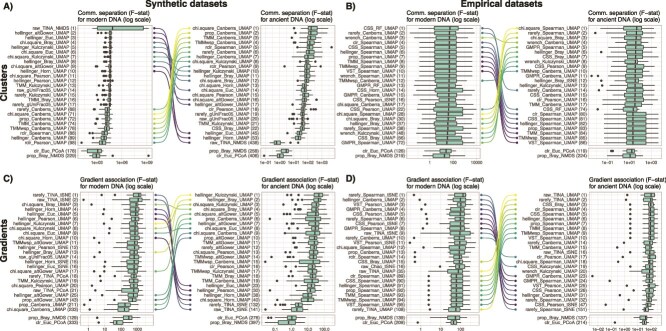
Performance of combinations of DNA read counts transformation, beta-diversity indices and ordinations for cluster separation in (A) synthetic and (B) empirical datasets, and for gradient association in (C) synthetic and (D) empirical datasets. Performance was measured with the F-statistic of PERMANOVA models (higher is better). The left panels show the F-statistic distributions for modern DNA datasets, whereas the right panels show the F-statistic distributions for simulated ancient DNA datasets. F-statistics were calculated using the Euclidean distances between pairs of samples in the ordinations as input of PERMANOVA models. Bumps charts connect identical combinations of methods that are among the 10 best approaches. Numbers between brackets indicate the ranked position of the combinations of count transformation, beta-diversity index and ordination based on the median F-statistic value.

Our results show that the combinations of count transformation, beta-diversity indices and ordinations performed differentially in detecting clusters and gradients in modern and ancient DNA datasets (Fig. 3, Tables S6–S9). The combinations that performed best for ancient DNA were less performant to detect clusters and gradients in modern DNA datasets, for both synthetic and empirical datasets. Interestingly, almost all combinations that performed well in detecting clusters and gradients in both modern and ancient DNA datasets included the UMAP ordination method. However, both UMAP and tSNE were ranked high for detecting gradients in modern synthetic and empirical DNA datasets (Fig. 3C and D). For both modern and ancient DNA, combinations that included the Chi-Square and Hellinger count transformation techniques were often ranked high in both synthetic and empirical datasets. Similarly, combinations that included the Canberra beta-diversity index for detecting clusters, and the Kulczynski or Bray–Curtis indices for detecting gradients, were often ranked at the top for synthetic datasets, while it was the Spearman index that performed well for empirical datasets, and to a lesser extent the Kulczynski or Bray–Curtis indices. The standard combinations of methods for beta-diversity analysis in microbial ecology (i.e. proportion, Bray–Curtis and NMDS or centered log ratio, Euclidean and PCoA) were ranked much lower for both synthetic and empirical and for both modern and ancient DNA datasets.

### Cross validation of supervised machine learning models and transfer functions performance

**Figure 4:**
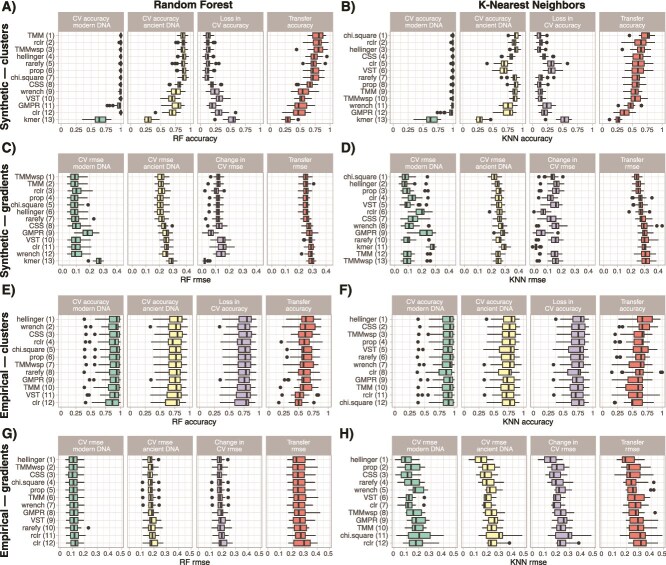
Performance of supervised models and transfer functions trained on composition matrices, i.e. using transformed DNA read counts as features, to infer the cluster of origin for synthetic (A and B) and empirical (E and F) datasets, and the ecological gradient value for synthetic (C and D) and empirical (G and H) datasets. We measured the performance of supervised models on both modern and ancient DNA datasets by cross validation (CV on two first panels, with a third panel showing the change in accuracy for clusters or in root mean square error for gradients between modern and ancient DNA). We finally measured the performance of transfer functions (the fourth panel, in red) trained only on modern samples to infer the cluster of origin and ecological gradient values on the ancient DNA samples. Numbers between brackets indicate the ranked performance of the count transformation techniques based on the median value of transfer accuracy-RMSE.

**Figure 5:**
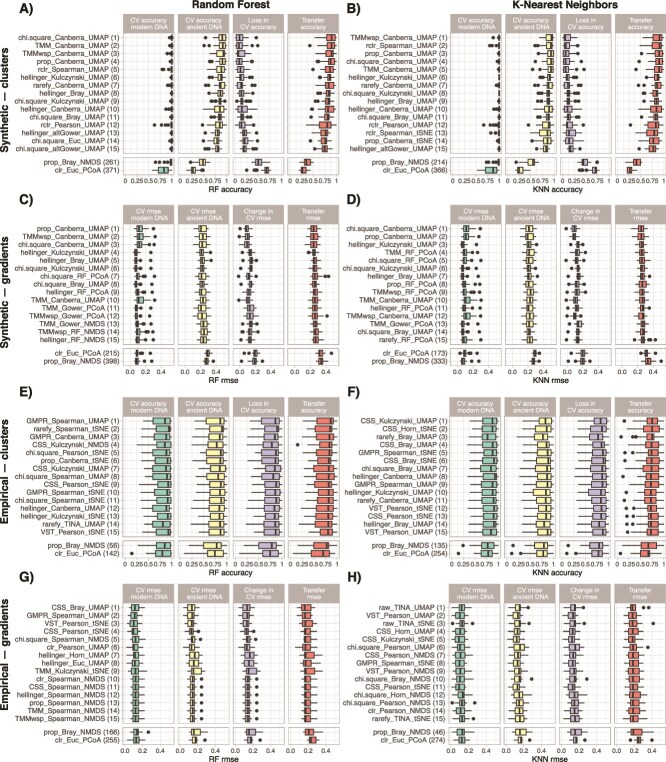
Performance of supervised models and transfer functions trained on ordinations embeddings, i.e. using samples coordinates on the ordination as features, to infer cluster of origin for synthetic (A and B) and empirical (E and F) datasets, and the ecological gradient value for synthetic (C and D) and empirical (G and H) datasets. We measured the performance of supervised models on both modern and ancient DNA datasets by cross validation (CV on two first panels, with a third panel showing the change in accuracy for clusters or in root mean square error for gradients between modern and ancient DNA). We finally measured the performance of transfer functions (the fourth panel, in red) trained only on modern samples to infer the cluster of origin and the ecological gradient values on the ancient DNA samples. Numbers between brackets indicate the ranked performance of the combinations of count transformation techniques, beta-diversity indices and ordinations methods based on the median value of transfer functions accuracy-RMSE.

We measured the performance of KNN and RF supervised models to predict the cluster of origin (classification accuracy), or the ecological gradient value (RMSE), for each sample on both modern and ancient DNA datasets using repeated 10-fold cross validations. Models were trained either on composition matrices (using the transformed DNA read counts as features, Fig. 4) or on ordination embeddings (using the samples coordinates in the two-dimensional space as features, Fig. 5).

For composition matrices, supervised models cross-validated on synthetic modern DNA datasets showed that count transformation techniques have only little effect on performance, except for the kmer transformation and to a lesser extent the GMPR transformation that led to reduced performance (Fig. 4A–D, Tables S10–S11). Performance on empirical datasets was mostly similar across the different count transformations techniques (Figs 4E–H, Tables S12–S13). Supervised models cross-validated on synthetic ancient DNA datasets show that the kmer, clr, GMPR, VST and wrench count transformation techniques are less performant than others, especially for cluster inference (Figs 4A–D). Again, this was not the case for ancient DNA datasets obtained from empirical datasets, where the count transformation technique showed little to no effect (Fig. 4E and F). However, the Hellinger transformation gave better results for empirical gradients (Fig. 4H). Finally, the count transformations techniques identified above also produced less performant transfer functions, especially for cluster inference in synthetic datasets (Figs 4A–D). The effect of count transformation techniques on transfer function performance on empirical datasets has only a limited effect (Figs 4E–H).

For ordination embeddings, supervised models cross-validated on both synthetic and empirical modern and ancient DNA datasets show that combinations of count transformation techniques, beta-diversity indices and, to a lesser extent, ordinations methods only slightly affect performance (Fig. 5, Tables S14–S17). The same was observed for the performance of transfer functions, although here the ordination method had a stronger effect on performance (Fig. S2). For cluster inferences, the combinations of methods including UMAP, and to a lesser extent tSNE ordinations, typically performed the best. For gradient inferences, combinations of methods including UMAP or PCoA performed best on synthetic datasets (Fig. 5C and D), whereas UMAP, tSNE or NMDS are usually the best for empirical datasets (Fig. 5G and H). The performance of transfer functions produced using standard combinations of methods in microbial ecology (i.e. proportion, Bray–Curtis and NMDS and center log ratio, Euclidean and PCoA) is much lower. For synthetic datasets, the combination of proportion, Bray–Curtis and NMDS was comparatively much better for clusters inference, whereas center log ratio, Euclidean and PCoA was better for gradients inference (Figs 5A–D). For empirical datasets, the performance of these combinations was similar for both cluster and gradient inference (Figs 5E–H).

**Figure 6:**
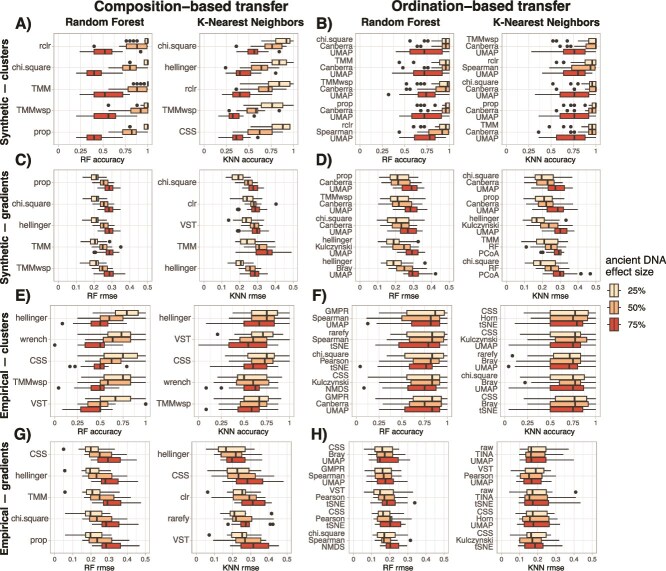
Performance of the top five composition-based and ordination-based transfer functions approaches in inferring cluster of origin for synthetic (A and B) and empirical (E and F) datasets, and the ecological gradient value for synthetic (C and D) and empirical (G and H) datasets, as a function of ancient DNA effect size.

**Figure 7:**
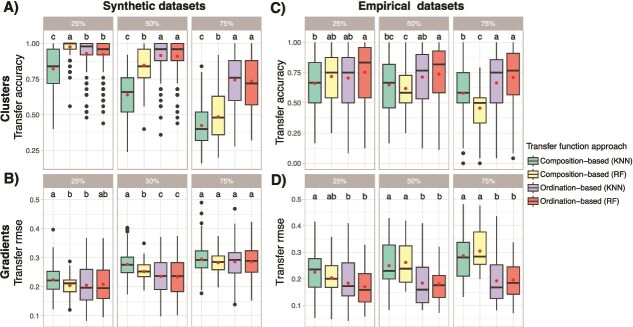
Aggregated performance metrics of the top five composition-based and ordination-based transfer functions approaches in inferring cluster of origin for synthetic (A) and empirical (C) datasets (transfer accuracy, higher is better), and the ecological gradient value for synthetic (B) and empirical (D) datasets (transfer RMSE, lower is better), as a function of ancient DNA effect size. We aggregated the performance metrics obtained from either random forest (RF) or the k-nearest neighbors (KNN) algorithms. We then compared the distributions of transfer functions accuracy-RMSE values using Tukey's honest significant difference to identify the best-performing transfer function approach at each level of ancient DNA effect size. Letters above the boxplots indicate groupings based on Tukey’s HSD: Transfer function approaches that share the same letter(s) are not significantly different from each other (*P* > .05).

Analyzing the five best composition and ordination-based transfer function approaches as a function of DNA effect size showed that the performance tends to drop as the ancient DNA effect sizes increases (Fig. 6). However, the performance of transfer functions calibrated on ordinations obtained from empirical datasets with the KNN algorithm is mostly similar at all ancient DNA effect sizes (Fig. 6F, H). We aggregated transfer accuracy and RMSE from these top five method combinations to compare the performance of composition-based and ordination-based transfer functions calibrated with either the random forest or k-nearest neighbors algorithms across the different levels of ancient DNA effect size. The best transfer function approach was not the same as the ancient DNA effect size increases (Fig. 7). For cluster and gradient inferences on both synthetic and empirical datasets, the performance of ordination-based transfer functions (using either RF or KNN) is usually better, especially as the ancient DNA effect size increases (Fig. 7A, C).

## Discussion

### Performance of beta-diversity measures from community dissimilarity matrices

Our benchmark of beta-diversity measures from community dissimilarity matrices shows that Pearson and Horn indices were among the best at detecting clusters and gradients in both modern and ancient DNA (Fig. 2). Although the combination of Chi-Square and Pearson index was ranked first for ancient DNA in synthetic datasets, this was not the case for modern DNA datasets. On empirical datasets, the approach combining Chi-Square and Pearson index was not among the top 25 combinations. Instead, combinations that included the TINA index were the best at detecting clusters on both modern and ancient DNA datasets, followed by combinations of methods that included the Person index (Fig. 2). Our results are in line with previous work on modern DNA datasets, including the study that introduced the TINA beta-diversity index and compared its performance with commonly used indices [32], and a benchmark of beta-diversity indices based on simulated and empirical datasets that identified the Pearson index as particularly performant in detecting gradients [27]. Here, the TINA index did not perform well at detecting clusters in ancient DNA versions of the synthetic datasets, whereas it was included in the best combinations for empirical datasets. This difference may stem from the fact that our empirical datasets had two to ten times more taxa than synthetic datasets, which only comprised 400 taxa. Since the TINA index includes a step to infer putative taxa interactions as part of the community dissimilarity measure, having more taxa may help detecting interactions and thus contribute to better separate clusters. Thus, we produced synthetic datasets with 2000 taxa and rerun the benchmark on these larger matrices. We then observed that the combination of rarefaction with the TINA index was the best at detecting clusters (Fig. S1). This supports the idea that the TINA index performs especially well in highly dimensional datasets, which is often the case in microbiome datasets. However, the TINA index includes the inference of an interaction network, which can be very computationally intensive. Since the Pearson and Horn indices were also identified as highly performant in detecting clusters and gradients, we recommend using these indices in highly dimensional datasets if computing power is limiting.

Finally, our results indicate that the most popular combinations of count transformation techniques and beta-diversity indices among microbial ecologists (i.e. taxa proportion and Bray–Curtis index, or centered log ratio and Euclidean distances) are never among the top 10 approaches for beta-diversity measures and perform significantly worse than other combinations tested here. This is also in line with previous work [27, 32]. The common use of these approaches may come from historical practices [54] or from a theoretical standpoint [26, 48],, and should be questioned based on the results presented here.

### Performance of beta-diversity measures and sample classification from community ordinations

Our results clearly show that combinations including UMAP, and to a lesser extent tSNE, almost always give the best results in detecting clusters and gradients in both modern and ancient DNA versions of all the tested datasets (Fig. 3). This was also the case for sample classification (or regression) with cross-validated supervised models (Fig. 5, Fig. S2). These ordinations methods consistently outperform the commonly used NMDS and PCoA that are currently the standard approach for DNA-based community ecology studies (Fig. 3, Fig. S2). Our results show that the choice of the ordination method has a much stronger effect on beta-diversity measures and sample classification compared to count transformation and beta-diversity index (Fig. S2). Combinations including UMAP and tSNE allowed extracting relevant ecological signals while compensating for technical noise due to ancient DNA signal introduced here, whereas combinations including NMDS and PCoA were sensitive to this noise. Indeed, NMDS ordinations were not separating the samples according to the DNA status (modern or ancient DNA), but were particularly prone to an increase in betadispersion (i.e. multivariate variance) as the ancient DNA effect size increases (Fig. S2 and S3). PCoA ordinations tended to separate samples based on the DNA status, rather than cluster of origin (Fig. S1 and S2). UMAP and tSNE were both able to extract the ecological signal while compensating for technical noise due to ancient DNA signal (Fig. S2). These differences likely contribute to explain the variations in performance for classifying (or regressing) samples by cross-validated supervised models in both modern and ancient DNA datasets (Fig. S2). These results are in line with previous work showing that UMAP proved superior to other ordinations methods in extracting relevant ecological signal in DNA-based microbiome datasets [35, 36]. Although not new to other disciplines utilizing high-throughput DNA sequencing technologies (e.g. for RNAseq analysis, [73]), UMAP and tSNE are currently underused in DNA-based community ecology studies. We therefore recommend these ordinations techniques in future studies, especially for ancient DNA data or in datasets with large variations in alpha diversity. Indeed, datasets with large variation in alpha diversity are prone to the horseshoe effect, where the samples are ordinated along a curve on the ordination space, because some samples may not share enough common taxa [74].

### Transfer functions based on ordination outperform the ones based on composition for sedaDNA datasets

The performance of transfer functions decreased in a broadly similar manner for both clusters and gradients inference as the ancient DNA effect size increased (Fig. 6 and 7), as expected given the incremental addition of technical noise in the data. However, transfer functions calibrated on ordinations were still sufficiently performant with ~50% of false negative taxa in synthetic datasets (Fig. 6B, D), and similarly performant across all ancient DNA effect sizes in empirical datasets (Fig. 6F, H). Inspecting the aggregated performance metrics obtained from the top five combinations of methods for composition and ordination-based transfer functions confirmed that using ordinations for calibrating a transfer function gives better clusters or gradients inferences, either using RF or KNN algorithms (Fig. 7). The gap in performance between composition and ordination-based approaches was increasing as the ancient DNA effect size increased, supporting the idea that ordinations (especially UMAP and tSNE) can compensate for technical noise due to ancient DNA signal. We thus recommend using transfer functions that are calibrated on samples coordinates obtained from community ordinations for *sed*aDNA studies.

### Limitations of our study

Here, we simulated ancient DNA signal on both “modern” synthetic and empirical datasets. We based these simulations on the idea that *sed*aDNA samples may have “lost” some or many taxa over time, due to DNA degradation once buried in the sediment. These taxa thus cannot be detected in *sed*aDNA datasets and constitutes false negative detections that introduces noise in data analysis. Since the environmental conditions and processes affecting DNA taphonomy in the sediment are not yet fully understood (but see [22]), our simulations may only partially capture these factors and thus might not fully reproduce realistic *sed*aDNA noise patterns in our datasets. Furthermore, we did not account for possible false positives taxa detections, due to “tag-jumps” that can cause incorrect assignment of sequences to samples in metabarcoding studies [75, 76]. Lastly, we simulated ancient DNA noise to our datasets, but we did not change the community structure in any way. Thus, our simulated ancient DNA datasets do not account for possible changes in taxa ecology over time and their resulted assembly into potentially different communities relative to modern conditions. To overcome these limitations, future work would need to produce *sed*aDNA datasets alongside conventional proxy data that are used for paleoceanographic or paleolimnological reconstructions.

## Conclusion and recommendations

Our study provides the first systematic benchmark of postprocessing workflows for beta-diversity measures and transfer functions using *sed*aDNA datasets. Based on our results, we recommend using the TINA, Pearson or Horn beta-diversity indices for beta-diversity measures from *sed*aDNA community dissimilarity matrices. For calibrating transfer functions to be applied on *sed*aDNA datasets, we recommend using the coordinates of samples from community ordinations (preferably obtained with UMAP or tSNE algorithms) rather than the composition matrices themselves, especially where DNA is expected to be highly degraded. By identifying method combinations that preserve the ecological signal under DNA degradation scenarios, our study contributes to the improving of paleoenvironmental reconstructions from *sed*aDNA data.

## Supplementary Material

Cordier_track_change_supplementary_ycaf230

Table_1-3_S1-17_revised_ycaf230

## Data Availability

The R code to reproduce the data simulation, analysis and figures is available on GitHub (https://github.com/trtcrd/Bench_sedaDNA). Empirical datasets from published papers are referenced in Table S1.
