# Supplementary material for: Benchmarking beta-diversity measures and transfer functions for sedimentary ancient DNA
Source: ISME Commun. 2025 Dec 6;5(1):ycaf230. doi: 10.1093/ismeco/ycaf230 (PMC12753309; doi:10.1093/ismeco/ycaf230)
Supplement: Cordier_track_change_supplementary_ycaf230 [file cordier_track_change_supplementary_ycaf230.docx]

**Supplementary figures**

*Figure S1: Performance of combinations of DNA read counts transformation and beta-diversity indices for cluster separation in synthetic datasets that contained 2000 taxa. Performance was measured with the F-statistic of PERMANOVA models (higher is better). The left panels show the F-statistic distributions for modern DNA datasets, whereas the right panels show the F-statistic distributions for simulated ancient DNA datasets. Bumps charts connect identical combinations of methods between modern and ancient DNA datasets that are among the 10 best approaches. Numbers between brackets indicate the ranked position of the combinations of count transformation and beta-diversity indices based on the median F-statistic value.*

**

*Figure S2: Performance of combinations of DNA read counts transformation, beta-diversity indices and ordinations methods for cluster separation (green panel), DNA status separation (yellow panel), change in betadispersion between DNA state (purple panel), cross validated accuracy on modern datasets (red panel), cross validated accuracy on ancient DNA datasets (blue panel) and transfer function accuracy (orange panel) in synthetic datasets. Only the top 20 combinations that gave best transfer accuracy per ordination method (UMAP, t-SNE, NMDS and PCoA) are shown.*

*Figure S3: Results obtained on one of the synthetic datasets with five clusters structure with the commonly used proportion transformation technique combined with the Bray-Curtis beta-diversity index and the NMDS ordination method. A) NMDS ordination obtained from the “modern” synthetic dataset. B) NMDS ordination of the same dataset after ancient DNA signal simulation. C) Distances of samples to DNA status centroid (i.e. betadispersion or multivariate variance) as function of normalized taxa (ASV on the axis) richness. D) Distribution of pairwise Bray-Curtis distances within groups (i.e within a given cluster) and between groups (between clusters), as well as the measured F-statistic from a PERMANOVA model. E) Same as D), but here the DNA status was used as factor. F) Proportion of correctly and wrongly classified samples to cluster of origin as a function of ancient DNA effect size. G) Receiver Operating Characteristic (ROC) curves for each individual clusters and overall measure of micro and macro-averages scores.*

*Figure S4: Results obtained on the same synthetic dataset as in Figure S3 with the commonly used center log ratio transformation technique combined with the Euclidean distance metric and the PCoA ordination method. A) PCoA ordination obtained from the “modern” synthetic dataset. B) PCoA ordination of the same dataset after ancient DNA signal simulation. C) Distances of samples to DNA status centroid (i.e. betadispersion or multivariate variance) as function of normalized taxa (ASV on the axis) richness. D) Distribution of pairwise Bray-Curtis distances within groups (i.e within a given cluster) and between groups (between clusters), as well as the measured F-statistic from a PERMANOVA model. E) Same as D), but here the DNA status was used as factor. F) Proportion of correctly and wrongly classified samples to cluster of origin as a function of ancient DNA effect size. G) Receiver Operating Characteristic (ROC) curves for each individual clusters and overall measure of micro and macro-averages scores.*

*Figure S5: Results obtained on the same synthetic dataset as in Figure S3 with the robust center log ratio transformation technique combined with the Spearman index as a measure of dissimilarity and the UMAP ordination method. A) UMAP ordination obtained from the “modern” synthetic dataset. B) UMAP ordination of the same dataset after ancient DNA signal simulation. C) Distances of samples to DNA status centroid (i.e. betadispersion or multivariate variance) as function of normalized taxa (ASV on the axis) richness. D) Distribution of pairwise Bray-Curtis distances within groups (i.e within a given cluster) and between groups (between clusters), as well as the measured F-statistic from a PERMANOVA model. E) Same as D), but here the DNA status was used as factor. F) Proportion of correctly and wrongly classified samples to cluster of origin as a function of ancient DNA effect size. G) Receiver Operating Characteristic (ROC) curves for each individual clusters and overall measure of micro and macro-averages scores.*
